# Site-Specific Risk Factors for Colorectal Cancer in a Korean Population

**DOI:** 10.1371/journal.pone.0023196

**Published:** 2011-08-10

**Authors:** Aesun Shin, Jungnam Joo, Jeongin Bak, Hye-Ryung Yang, Jeongseon Kim, Sohee Park, Byung-Ho Nam

**Affiliations:** 1 Cancer Epidemiology Branch, National Cancer Center, Goyang-si, Republic of Korea; 2 Cancer Biostatistics Branch, National Cancer Center, Goyang-si, Republic of Korea; National Taiwan University Hosipital, Taiwan

## Abstract

We investigated the association of colorectal cancer risk factors with different colorectal cancer subsites to assess etiological differences for cancers of the proximal colon, distal colon, and rectum. Included in this study were 869,725 men and 395,501 women who participated in a health examination provided by the Korean National Health System between 1996 and 1997. During up to 7 years of follow-up, 4,144 incident colorectal cancer cases were detected (3,051 men and 1,093 women). Greater height was associated with elevated risk for distal colon cancer and rectal cancer in both men and women. Family history of cancer was associated with higher risk for cancers of the proximal colon in men and distal colon in both men and women. Frequent alcohol consumption and consuming high amounts of alcohol were associated with elevated risk for distal colon cancer in men and higher risk for rectal cancer in women. Frequent meat consumption was associated with risk for proximal colon cancer in men and for rectal cancer in women. Our findings suggest that risk factors for colorectal cancer are different by subsites of colon and rectum, as well as by sex.

## Introduction

Colorectal cancer incidence and mortality has been increasing rapidly in Korea during last few decades. According to the Korean Central Cancer Registry, colorectal cancer is the third most common incident cancer with age-adjusted incidences of 47.0 per 100,000 for men and 25.6 for women in 2008 [1]. The annual percent changes in colorectal cancer incidence were 6.9% in men and 5.2% in women between 1999 and 2008 [1], and the incidence and mortality rates of colorectal cancer are expected to increase [2]. The proximal and distal colon and rectum have different embryologic origins, morphologic appearance of normal mucosa, metabolizing enzyme activity, physiological function, fecal composition, bile acid metabolism, and intestinal transit times [3], [4]. Recent clinicopathologic and molecular studies of colorectal cancer have suggested that there are subtypes based on tumor methylation status and DNA microsatellite instability (MSI) that exhibit different characteristics in patients, such as differences in tumor locations [5], [6]. Specifically, CpG island methylator phenotype (CIMP)-high tumors are mainly found in the proximal colon, whereas no-CIMP and microsatellite-stable (MSS) tumors are mainly located in the distal colon [5], [6]. Although classification of colorectal cancer based on anatomical site of origin is too crude to distinguish phenotypically distinct colorectal cancer subgroups, it is still useful especially in large scale cohort studies, in which detailed molecular classification of tumors may not be available for analysis.

Several cohort studies have been conducted to assess risk factor profiles for cancers in different subsites of the colon/rectum in Asian populations [7], [8], [9], and the results have been inconclusive. The objective of the current study was to assess the association between different subsites of colorectal cancers and colorectal risk factors based on a large health insurance study conducted in Korea, where the incidence of colorectal cancer is increasing steadily.

## Materials and Methods

### Study population

Included in this study were men and women who were beneficiaries or dependent family members of the insured of the Korean National Health System (KNHS), a major institution for the nationwide health insurance system in Korea, and who participated in a health examination provided by the KNHS between 1996 and 1997. This study involved routinely collected medical data, hence participant's consent was not required and the study protocol was approved by the Institutional Review Board of the National Cancer Center (IRB no. NCCNCS-09-305).

Participants were asked to fill out a questionnaire on alcohol consumption, cigarette smoking habits, regular exercise, family history of cancer, dietary preferences, and consumption frequency of meats. Height and weight were measured during the health examination. A total of 1,265,226 participants (869,725 men and 395,501 women), aged 30 to 80 years, who participated in the health examination, without previous history of cancer, and with no missing information for any of the major risk factor variables including height, weight, fasting serum glucose, total serum cholesterol, family history of cancer, cigarette smoking status (current/ex-/non-smokers), and alcohol consumption frequency) were included in the final analysis.

### Colorectal cancer ascertainment

Cancer occurrence was ascertained from the Korean Central Cancer Registry data, and death information from the Korean National Statistical Office up to December 2003. Subsites of colorectal cancer were categorized by the International Classification of Disease 10th edition (ICD-10) codes as follows: proximal colon (C180–C185), distal colon (C186–C187), and rectum (C19–C20). Cancers with an overlapping lesion of the colon (C188) or that were not otherwise specified (C189) were excluded from the analysis.

### Statistical analysis

The chi-square test was used for comparing risk factor distributions between non-case groups and colorectal cancer groups. The Cox proportional hazard model was used to estimate the age-adjusted hazard ratios (HR) of the risk factors and their 95% confidence intervals (CI). Age-adjusted hazard ratios for exposure variables were calculated to compare hazard ratios of each variable across cancer sites. Colorectal cancer cases detected at subsites other than the site of interest were censored at the time of diagnosis in survival analyses. P for trends in the hazard ratios were calculated using the order of each risk factor category as a categorical variable. The ‘unknown’ category was not included in trend analyses. The likelihood ratio tests were conducted to examine whether the effects of the potential risk factors were the same across different cancer subsites. When the likelihood ratio tests showed significant difference, Wald chi-square (1 d. f.) tests based on the coefficients and corresponding standard errors estimated from the Cox regression model were constructed to further single out the difference between any pairs of cancer subsites [10]. All analyses were conducted using SAS software version 9.1 (SAS Institute Inc, Cary, NC).

## Results

During up to 7 years of follow-up, 4,144 colorectal cancer incidents were detected (3,051 in men and 1,093 in women; Table 1 and 2). Among men, 536 proximal colon cancers, 751 distal colon cancers, and 1,535 rectal cancers were found. Among women, 236 proximal colon cancers, 225 distal colon cancers, and 451 rectal cancers were found. Cases with overlapping lesions in the colon or whose cancers were not otherwise specified lesions were excluded (229 men and 81 women).

**Table 1:** Risk factor distributions between colorectal cancer patients and colorectal cancer-free participants, men, N(%).

	Colorectal cancer-free participants (N = 866,674)	Proximal colon cancer cases (N = 536)	Distal colon cancer cases (N = 751)	Rectal cancer cases (N = 1,535)
Age*	45.2 (10.5)	54.1 (10.9)	55.9 (9.7)	54.7 (10.0)
*P*		<0.001	<0.001	<0.001
Height (cm)				
≤165	266,295 (30.7)	211 (39.4)	275 (36.6)	571 (37.2)
165.1–168	220,512 (25.4)	136 (25.4)	203 (27.0)	432 (28.1)
168.1–172	181,971 (21)	97 (18.1)	145 (19.3)	280 (18.2)
>172	197,896 (22.8)	92 (17.2)	128 (17.0)	252 (16.4)
*P*		<0.001	<0.001	<0.001
BMI (kg/m^2^)				
<18.5	21,903 (2.5)	19 (3.5)	18 (2.4)	57 (3.7)
18.5–22.9	361,766 (41.7)	210 (39.2)	259 (34.5)	609 (39.7)
23.0–24.9	244,341 (28.2)	156 (29.1)	213 (28.4)	439 (28.6)
≥25	238,664 (27.5)	151 (28.2)	261 (34.8)	430 (28.0)
*P*		0.354	<0.001	0.016
Fasting glucose (mg/dl)				
<126	813,594 (93.9)	506 (94.4)	669 (89.1)	1,376 (89.6)
≥126	53,080 (6.1)	30 (5.6)	82 (10.9)	159 (10.4)
*P*		0.602	<0.001	<0.001
Serum total cholesterol (mg/dl)				
≤200	554,690 (64.0)	333 (62.1)	448 (59.7)	876 (57.1)
201–239	231,894 (26.8)	163 (30.4)	211 (28.1)	487 (31.7)
≥240	80,090 (9.2)	40 (7.5)	92 (12.3)	172 (11.2)
*P*		0.091	0.007	<0.001
Family history of cancer				
No	708,382 (81.7)	421 (78.5)	594 (79.1)	1,272 (82.9)
Yes	158,292 (18.3)	115 (21.5)	157 (20.9)	263 (17.1)
*P*		0.056	0.061	0.250
Meat consumption frequency (per week)				
≤1 time	400,504 (46.2)	222 (41.4)	336 (44.7)	705 (45.9)
2–3 times	414,930 (47.9)	269 (50.2)	344 (45.8)	702 (45.7)
≥4 times	44,439 (5.1)	43 (8.0)	64 (8.5)	112 (7.3)
Unknown	6,801 (0.8)	2 (0.4)	7 (0.9)	16 (1.0)
*P*		0.004	<0.001	0.001
Cigarette smoking habits				
Never	245,000 (28.3)	170 (31.7)	232 (30.9)	475 (30.9)
Former	129,259 (14.9)	94 (17.5)	184 (24.5)	288 (18.8)
Current	492,415 (56.8)	272 (50.8)	335 (44.6)	772 (50.3)
*P*		0.017	<0.001	<0.001
Alcohol consumption frequency				
None	257,802 (29.8)	176 (32.8)	230 (30.6)	525 (34.2)
2–3 times/month	181,201 (20.9)	93 (17.4)	119 (15.9)	280 (18.2)
1–2 times/week	248,629 (28.7)	137 (25.6)	194 (25.8)	348 (22.7)
3–4 times/week	117,681 (13.6)	70 (13.1)	130 (17.3)	218 (14.2)
Almost everyday	61,361 (7.1)	60 (11.2)	78 (10.4)	164 (10.7)
*P*		0.001	<0.001	<0.001
Alcohol consumption amount				
None	257,802 (29.8)	176 (32.8)	230 (30.6)	525 (34.2)
Less than half bottle of Korean distilled spirits	192,937 (22.3)	125 (23.3)	183 (24.4)	413 (26.9)
One bottle of Korean distilled spirits	285,406 (32.9)	172 (32.1)	251 (33.4)	416 (27.1)
More than one bottle	126,440 (14.6)	61 (11.4)	86 (11.5)	171 (11.1)
Unknown	4,089 (0.5)	2 (0.4)	1 (0.1)	10 (0.7)
*P*		0.203	0.072	<0.001

*mean (standard deviation), P-values were derived from chi-square test compared to colorectal cancer-free participants, except for p-values for age, which were derived from t-test.

**Table 2:** Risk factor distributions between colorectal cancer patients and colorectal cancer-free participants, women, N(%).

	Colorectal cancer-free participants (N = 394,408)	Proximal colon cancer cases (N = 236)	Distal colon cancer cases (N = 225)	Rectal cancer cases (N = 551)
Age*	49.1 (11.2)	58.8 (10.4)	55.3 (10.3)	57.1 (10.3)
*P*		<0.001	<0.001	<0.001
Height (cm)				
≤151	107,212 (27.2)	89 (37.7)	68 (30.2)	184 (33.4)
151.1–155	104,693 (26.5)	65 (27.5)	65 (28.9)	160 (29.0)
155.1–159	98,770 (25.0)	52 (22.0)	56 (24.9)	117 (21.2)
>159	83,733 (21.2)	30 (12.7)	36 (16.0)	90 (16.3)
*P*		<0.001	0.250	<0.001
BMI (kg/m^2^)				
<18.5	16,278 (4.1)	9 (3.8)	10 (4.4)	17 (3.1)
18.5–22.9	172,042 (43.6)	76 (32.2)	75 (33.3)	209 (37.9)
23.0–24.9	94,844 (24.1)	54 (22.9)	63 (28.0)	135 (24.5)
≥25	111,244 (28.2)	97 (41.1)	77 (34.2)	190 (34.5)
*P*		<0.001	0.020	0.004
Fasting glucose (mg/dl)				
<126	374,756 (95.0)	215 (91.1)	213 (94.7)	506 (91.8)
≥126	19,652 (5.0)	21 (8.9)	12 (5.3)	45 (8.2)
*P*		0.006	0.814	0.001
Serum total cholesterol (mg/dl)				
≤200	239,520 (60.7)	111 (47.0)	109 (48.4)	293 (53.2)
201–239	108,707 (27.6)	88 (37.3)	82 (36.4)	176 (31.9)
≥240	46,181 (11.7)	37 (15.7)	34 (15.1)	82 (14.9)
*P*		<0.001	0.001	0.001
Family history of cancer				
No	318,999 (80.9)	196 (83.1)	168 (74.7)	452 (82.0)
Yes	75,409 (19.1)	40 (17.0)	57 (25.3)	99 (18.0)
*P*		0.396	0.018	0.491
Meat consumption frequency (per week)				
≤1 time	228,873 (58.0)	133 (56.4)	125 (55.6)	303 (55.0)
2–3 times	136,965 (34.7)	71 (30.1)	78 (34.7)	194 (35.2)
≥4 times	23,410 (5.9)	24 (10.2)	16 (7.1)	44 (8.0)
Unknown	5,160 (1.3)	8 (3.4)	6 (2.7)	10 (1.8)
*P*		0.001	0.271	0.119
Cigarette smoking habits				
Never	371,859 (94.3)	217 (92.0)	206 (91.6)	502 (91.1)
Former	5,042 (1.3)	6 (2.5)	3 (1.3)	10 (1.8)
Current	17,507 (4.4)	13 (5.5)	16 (7.1)	39 (7.1)
*P*		0.159	0.151	0.005
Alcohol consumption frequency				
None	331,580 (84.1)	206 (87.3)	201 (89.3)	446 (80.9)
2–3 times/month	37,951 (9.6)	19 (8.1)	17 (7.6)	54 (9.8)
1–2 times/week	17,891 (4.5)	5 (2.1)	5 (2.2)	26 (4.7)
3–4 times/week	3,901 (1.0)	5 (2.1)	1 (0.4)	9 (1.6)
Almost everyday	3,085 (0.8)	1 (0.4)	1 (0.4)	16 (2.9)
*P*		0.118	0.249	<0.001
Alcohol consumption amount				
None	331,580 (84.1)	206 (87.3)	201 (89.3)	446 (80.9)
Less than half bottle of Korean distilled spirits	49,975 (12.7)	23 (9.8)	21 (9.3)	85 (15.4)
More than one bottle of Korean distilled spirits	8,701 (2.2)	6 (2.5)	2 (0.9)	15 (2.7)
Unknown	4,152 (1.1)	1 (0.4)	1 (0.4)	5 (0.9)
*P*		0.409	0.152	0.193

*ean (standard deviation), P-values were derived from chi-square test compared to colorectal cancer-free participants, except for p-values for age, which were derived from t-test.

Those who developed colorectal cancers were more likely to be older than those who did not (p<0.001) both in men and women. After adjusting for age, height was associated with elevated risk for distal colon cancer in men (*P-_trend_* = 0.003; Table 3). Similarly, height was associated with increased risk for rectal cancer in women (*P-_trend_* = 0.004; Table 4) and marginally elevated risk for rectal cancer in men (*P-_trend_* = 0.052). Higher BMI was associated with elevated risk for distal colon cancer in men (*P-_trend_*<0.001) and with marginally elevated risk for proximal colon cancer in women (*P-_trend_* = 0.057). High serum total cholesterol was associated with high risk for rectal cancer (*P-_trend_* = 0.001), and marginal risk for distal colon cancer (*P-_trend_* = 0.079) in men. Whereas a serum fasting glucose level of 126 mg/dl or higher was associated with higher risk for distal colon cancer (HR = 1.3, 95% CI: 1.1, 1.7) and rectal cancer (HR = 1.3, 95% CI: 1.1, 1.5) in men, it was also associated with lower risk for proximal colon cancer (HR = 0.7, 95% CI: 0.5, 1.0). On the other hand, no significant association was observed between serum total cholesterol or fasting glucose and colorectal cancer in women. Family history of cancer was associated with higher risk for cancers of the proximal colon (hazard ratio (HR) = 1.4, 95% CI: 1.1, 1.7) in men and distal colon in both men (HR = 1.4, 95% CI: 1.2, 1.7) and women (HR = 1.6, 95% CI: 1.2, 2.2).

**Table 3:** Association between risk factors and risk of cancer of the proximal colon, distal colon and rectum in men, hazard ratios (95% confidence intervals).

	Proximal colon cancer	Distal colon cancer*	Rectal cancer
Age (/year)	1.1 (1.1, 1.1)	1.1 (1.1, 1.1)	1.1 (1.1, 1.1)
Height (cm)			
≤165	1.0 (reference)	1.0 (reference)	1.0 (reference)
165.1–168	1.0 (0.8, 1.3)	1.3 (1.1, 1.5)	1.2 (1.1, 1.4)
168.1–172	1.1 (0.8, 1.3)	1.4 (1.1, 1.7)	1.2 (1.0, 1.4)
>172	1.1 (0.8, 1.4)	1.3 (1.1, 1.6)	1.1 (1.0, 1.3)
*P-trend*	0.567	0.003	0.052
BMI (kg/m^2^)			
<18.5	1.1 (0.7, 1.8)	0.8 (0.5, 1.2)	1.1 (0.8, 1.4)
18.5–22.9	1.0 (reference)	1.0 (reference)	1.0 (reference)
23.0–24.9	1.1 (0.9, 1.4)	1.3 (1.1, 1.5)	1.1 (1.0, 1.2)
≥25	1.1 (0.9, 1.4)	1.6 (1.4, 2.0)	1.1 (1.0, 1.3)
*P-trend*	0.299	<0.001	0.085
Fasting glucose (mg/dl)			
<126	1.0 (reference)	1.0 (reference)	1.0 (reference)
≥126	0.7 (0.5, 1.0)	1.3 (1.1, 1.7)	1.3 (1.1, 1.5)
*P*	0.030	0.020	0.004
Serum total cholesterol (mg/dl)			
≤200	1.0 (reference)	1.0 (reference)	1.0 (reference)
201–239	1.1 (0.9, 1.3)	1.0 (0.9, 1.2)	1.2 (1.1, 1.4)
≥240	0.7 (0.5, 1.0)	1.3 (1.0, 1.6)	1.2 (1.0, 1.4)
*P-trend*	0.333	0.079	0.001
Family history of cancer			
No	1.0 (reference)	1.0 (reference)	1.0 (reference)
Yes	1.4 (1.1, 1.7)	1.4 (1.2, 1.7)	1.1 (0.9, 1.2)
*P*	0.002	<0.001	0.336
Meat consumption frequency (per week)			
≤1 time	1.0 (reference)	1.0 (reference)	1.0 (reference)
2–3 times	1.3 (1.1, 1.5)	1.1 (0.9, 1.3)	1.0 (0.9, 1.2)
≥4 times	1.4 (1.0, 1.9)	1.3 (1.0, 1.7)	1.1 (0.9, 1.3)
Unknown	0.4 (0.1, 1.4)	0.8 (0.4, 1.6)	0.9 (0.5, 1.4)
*P-trend*	0.005	0.076	0.280
Cigarette smoking habits			
Never	1.0 (reference)	1.0 (reference)	1.0 (reference)
Former	1.0 (0.8, 1.3)	1.4 (1.2, 1.7)	1.1 (1.0, 1.3)
Current	1.0 (0.8, 1.2)	0.9 (0.8, 1.1)	1.0 (0.9, 1.1)
*P-trend*	0.806	0.161	0.975
Alcohol consumption frequency			
None	1.0 (reference)	1.0 (reference)	1.0 (reference)
2–3 times/month	1.1 (0.8, 1.4)	1.1 (0.9, 1.4)	1.1 (0.9, 1.3)
1–2 times/week	1.1 (0.9, 1.4)	1.4 (1.1, 1.7)	1.0 (0.9, 1.2)
3–4 times/week	1.1 (0.8, 1.4)	1.7 (1.3, 2.1)	1.2 (1.0, 1.4)
Almost everyday	1.2 (0.9, 1.7)	1.2 (0.9, 1.5)	1.1 (0.9, 1.3)
*P-trend*	0.136	<0.001	0.113
Alcohol consumption amount			
None	1.0 (reference)	1.0 (reference)	1.0 (reference)
Less than half bottle	1.0 (0.8, 1.2)	1.1 (0.9, 1.3)	1.1 (0.9, 1.2)
One bottle of Korean distilled spirits	1.3 (1.0, 1.6)	1.6 (1.3, 1.9)	1.1 (0.9, 1.2)
More than one bottle of Korean distilled spirits	1.2 (0.9, 1.6)	1.5 (1.2, 2.0)	1.2 (1.0, 1.4)
Unknown	0.6 (0.1, 2.3)	0.2 (0.0, 1.5)	0.9 (0.5, 1.8)
*P-trend*	0.024	<0.001	0.109

*HR: hazard ratio, CI: confidence interval. Hazard ratios were adjusted for age. *P-trends* were calculated using the order of categories as a continuous variable in the proportional hazard models.

**Table 4:** Association between risk factors and cancer of the proximal colon, distal colon, and rectum in women, hazard ratios (95% confidence intervals).

	Proximal colon cancer	Distal colon cancer*	Rectal cancer
Age (/year)	1.1 (1.1, 1.1)	1.1 (1.0, 1.1)	1.1 (1.1, 1.1)
Height (cm)			
≤151	1.0 (reference)	1.0 (reference)	1.0 (reference)
151.1–155	1.2 (0.9, 1.7)	1.4 (1.0, 1.9)	1.4 (1.1, 1.7)
155.1–159	1.3 (0.9 ,1.9)	1.5 (1.0, 2.2)	1.3 (1.0, 1.7)
>159	1.2 (0.8, 1.9)	1.3 (0.9, 2.1)	1.5 (1.1, 2.0)
*P-trend*	0.214	0.102	0.004
BMI (kg/m^2^)			
<18.5	1.1 (0.6, 2.3)	1.4 (0.7, 2.7)	0.8 (0.5, 1.3)
18.5–22.9	1.0 (reference)	1.0 (reference)	1.0 (reference)
23.0–24.9	1.1 (0.8, 1.6)	1.3 (1.0, 1.9)	1.0 (0.8, 1.3)
≥25	1.5 (1.1, 2.0)	1.3 (0.9, 1.8)	1.1 (0.9, 1.3)
*P-trend*	0.057	0.234	0.318
Fasting glucose (mg/dl)			
<126	1.0 (reference)	1.0 (reference)	1.0 (reference)
≥126	1.2 (0.8, 1.9)	0.8 (0.4, 1.4)	1.2 (0.9, 1.6)
*P*	0.424	0.437	0.327
Serum total cholesterol (mg/dl)			
≤200	1.0 (reference)	1.0 (reference)	1.0 (reference)
201–239	1.3 (1.0, 1.7)	1.3 (1.0, 1.8)	1.0 (0.8, 1.2)
≥240	1.1 (0.7, 1.6)	1.2 (0.8, 1.7)	1.0 (0.8, 1.2)
*P-trend*	0.355	0.170	0.820
Family history of cancer			
No	1.0 (reference)	1.0 (reference)	1.0 (reference)
Yes	1.1 (0.7, 1.5)	1.6 (1.2, 2.2)	1.1 (0.9, 1.3)
*P*	0.774	0.002	0.481
Meat consumption frequency (per week)			
≤1 time	1.0 (reference)	1.0 (reference)	1.0 (reference)
2–3 times	1.0 (0.7, 1.3)	1.1 (0.9, 1.5)	1.2 (1.0, 1.4)
≥4 times	1.7 (1.1, 2.7)	1.3 (0.7, 2.1)	1.4 (1.0, 1.9)
Unknown	2.2 (1.1, 4.5)	1.9 (0.8, 4.3)	1.2 (0.7 2.3)
*P-trend*	0.097	0.270	0.020
Cigarette smoking habits			
Never	1.0 (reference)	1.0 (reference)	1.0 (reference)
Former	1.1 (0.5, 2.6)	0.7 (0.2, 2.4)	0.9 (0.5, 1.7)
Current	0.7 (0.4, 1.2)	1.1 (0.7, 1.9)	1.0 (0.7, 1.4)
*P-trend*	0.252	0.767	0.967
Alcohol consumption frequency			
None	1.0 (reference)	1.0 (reference)	1.0 (reference)
2–3 times/month	1.0 (0.6, 1.6)	0.9 (0.5, 1.4)	1.3 (1.0, 1.7)
1–2 times/week	0.5 (0.2, 1.2)	0.5 (0.2, 1.2)	1.2 (0.8, 1.7)
3–4 times/week	1.8 (0.7, 4.4)	0.4 (0.1, 2.8)	1.5 (0.8, 3.0)
Almost everyday	0.4 (0.1, 2.6)	0.4 (0.1, 3.0)	2.8 (1.7, 4.7)
*P-trend*	0.372	0.042	<0.001
Alcohol consumption amount			
None	1.0 (reference)	1.0 (reference)	1.0 (reference)
Less than half bottle of Korean distilled spirits	0.8 (0.5, 1.3)	0.7 (0.5, 1.2)	1.4 (1.1, 1.7)
More than one bottle of Korean distilled spirits	1.6 (0.7, 3.5)	0.5 (0.1, 1.9)	1.7 (1.0, 2.8)
Unknown	0.4 (0.1, 2.9)	0.4 (0.1, 3.0)	0.9 (0.4, 2.3)
*P-trend*	0.941	0.094	0.001

*HR: hazard ratio, CI: confidence interval. Hazard ratios were adjusted for age. *P-trends* were calculated using the order of categories as a continuous variable in the proportional hazard models.

Frequent meat consumption was associated with risk for proximal colon cancer in men (*P-_trend_* = 0.005) and with risk for rectal cancer in women (*P-_trend_* = 0.02). Frequent alcohol consumption and consuming greater amounts of alcohol were both associated with elevated risk for distal colon cancer in men (*P-_trend_*<0.001, respectively), whereas women who frequently consumed alcohol or who consumed greater amounts of alcohol had higher risk for rectal cancer (*P-_trend_*<0.001, respectively). Current cigarette smoking was not associated with colorectal cancer risk in our study population, except that former smoker had 1.4-fold increased risk of distal cancer in men (95% CI: 1.2, 1.7).

In men, the likelihood ratio results indicates the effects of current cigarette smoking , fasting glucose level, alcohol consumption amount, BMI , serum total cholesterol and family history of cancer (marginal) vary depending on colorectal cancer subsites (Table 5). A serum fasting glucose level of 126 mg/dl or higher was a significant risk factor for distal colon cancer and rectal cancer, whereas it showed a significant protective effect in proximal colon cancer in our data. High alcohol consumption amount and high BMI (> = 25) were a significant risk factor only for distal colon cancer. Similarly, the effect of family history of any cancer was significant only for colon cancers.

**Table 5:** Results of the likelihood ratio statistics for testing the differences of each risk factor across colorectal cancer subsites.

	Men			Women		
	*df*	*Chi-square*	*P*	*df*	*Chi-square*	*P*
Height (cm)	8	5.817	0.668	8	9.875	0.274
BMI (kg/m^2^)	8	18.458	0.018	8	14.271	0.075
Fasting glucose (mg/dl)	4	13.847	0.008	4	9.808	0.044
Serum total cholesterol (mg/dl)	6	12.976	0.043	6	11.884	0.065
Family history of cancer	4	8.993	0.061	4	13.321	0.010
Meat consumption frequency (per week)	8	7.493	0.484	8	12.162	0.144
Cigarette smoking habits	6	24.89	<0.001	6	10.403	0.109
Alcohol consumption frequency	10	14.823	0.139	10	28.792	0.001
Alcohol consumption amount	10	22.152	0.014	8	21.353	0.006

In women, alcohol consumption (both frequency and amount), family history of cancer and serum glucose level were found to be different. Alcohol consumption was a significant risk factor only for rectal cancer, but not for colon cancers. Family history of cancer was associated significantly only with distal colon cancer.

## Discussion

In this large-scale cohort study, we consistently found significant positive associations between height and risk of rectal cancer in both men and women. Higher BMI was associated with increased risk for distal colon cancer in men and for proximal colon cancer in women. In addition, family history of cancer was associated with increased risk for distal colon cancer in both men and women and for proximal colon cancer in men. Frequent meat consumption was associated with increased risk for proximal colon cancer in men and that for rectal cancer in women, and alcohol consumption was associated with increased risk for distal colon cancer in men and for rectal cancer in women.

Greater height was associated with elevated risk for distal colon cancer and for rectal cancer in both men and women. Positive association between height and colorectal cancer risk in men is consistent with a previous study using a similar health insurance database in a Korean population [11]. However, the same study observed no association between height and female rectal cancer risk [11], which is not consistent with our result. Further analysis with a longer follow-up period to ensure statistical power for each of the subsites of colorectal cancer is warranted to clarify the role of height in the carcinogenesis of each subsite.

Obesity has been consistently associated with increased risk for colon cancer in men, however, the association has been inconsistent in women [12]. A meta-analysis of prospective studies showed that an increase in BMI was associated with increased risk of colon cancer in both men and women, although the association was stronger in men. In addition, BMI was positively association with rectal cancer risk in men, but not in women [12]. The summary relative risks for colon and rectal cancer were not differ by geographic regions where studies were conducted [12]. Positive associations between BMI and risk for colon and rectal cancer in men and risk for colon cancer in postmenopausal women have been found in previous Korean health insurance studies [13], [14]. Our results suggest the possibility that associations between colon cancer and obesity can be mainly attributed to distal colon cancer in men and to proximal colon cancer in women.

Metabolic risk factors may contribute to carcinogenesis through affecting insulin resistance, aromatase activity, adipokine production, angiogenesis, glucose utilization, oxidative stress, and DNA damage [15]. Diabetes mellitus has been associated with a 30% elevated risk for colorectal cancer in a meta-analysis, and the associations did not differ statistically by sex or by cancer subsite [16]. Our finding of decreased risk for proximal colon cancer among subjects with serum glucose level of 126 mg/dL or higher is an unexpected result. However, due to the limited information on diabetes drug use, and relatively small number of proximal colon cases with serum glucose level of 126 mg/dL or higher, these results should be interpreted with caution.

In the Alpha-Tocopherol Beta-Carotene Prevention Study, high serum total cholesterol was associated with decreased risk for rectal cancer and was not associated with colon cancer risk [17], whereas in a prospective study of Japanese-American men, high serum total cholesterol was associated with decreased risk for colon cancer and was not associated with rectal cancer risk [18]. In the Physicians' Health Study, no association between serum total cholesterol level and risk for colorectal cancer was observed [19]. In contrast, history of hypercholesterolemia has been related to high risk for colorectal cancer in men [20]. Although serum total cholesterol alone is not an optimal marker for dyslipidemia [21], our results suggest a differential etiologic role for hyperlipidemia in cancer subsites (i.e., colon and rectum) in men. Our finding of no association between serum total cholesterol or fasting glucose level and colorectal cancer risk in women is consistent with previous prospective studies, which have shown no association between metabolic risk factors and colorectal cancer risk in women [20], [22], [23], [24], [25].

Although family history of colorectal cancer is a well-defined risk factor for colorectal cancer, little is known about the role of family history of extracolonic cancers. However, cancer of the proximal colon has been found to be more frequent in patients with two or more tumors in a first-degree relative among registered colorectal cancer patients [26], which is consistent with our results.

In a meta-analysis of 15 prospective studies on meat intake, consumption of red meat and processed meat was positively associated with risk of both colon and rectal cancer [27]. The association with red meat was more pronounced for rectal cancer than for colon cancer. In three studies that reported results for subsites of the colon, high consumption of processed meat was associated with an increased risk of distal colon cancer, but not of proximal colon cancer. High consumption of red meat was not differentially associated with the risk of proximal or distal colon cancers, although the risk estimate for distal colon cancer was slightly higher than for proximal colon cancer [27]. Although the question on meat in our study covered only frequency of consumption, we observed positive associations between frequent meat consumption and proximal colon cancer risk in men and risk for proximal colon cancer and rectal cancer in women. Recently, the International Agency for Research on Cancer (IARC) recognized cigarette smoking as a risk factor for colorectal cancer, a tumor site for which there is sufficient evidence that tobacco smoking causes cancer [28]. Two meta-analyses have confirmed the association between cigarette smoking and increased risk for colorectal cancers, and the association was stronger for rectal cancer than for colon cancer [29], [30]. In contrast, we observed no association between cigarette smoking and colorectal cancer risk in our study population.

A pooled analysis of eight cohort studies showed that positive associations between alcohol intake and colorectal cancer [31]. A pooled analysis of five Japanese cohort studies also suggested positive associations between alcohol intake and risk of colon and rectal cancers in both men and women [8]. The association was more prominent for rectal cancer in some studies [32], [33], but not in other studies [34], [35], [36]. In the Health Professionals Follow-Up Study and the Nurses' Health Study, alcohol intake was associated with increased risk for colon cancer only in men, whereas no apparent associations were found between colon or rectal cancers and alcohol intake in women [36]. In the Netherlands Cohort Study, high amounts of alcohol consumption was associated with increased risk for rectal cancer in men, whereas it was associated with risk for proximal and rectosigmoid cancers in women [34]. Different types of alcoholic beverages appear to have different effects on the association with colorectal cancer risk. Beer seems to increase the risk for colorectal cancer [32], [36], whereas wine seems to decrease the risk [33].

The strength of the current study includes a large sample size and completeness of cancer follow-up by data linkage to cancer registration. Weight and height, which are important risk factors for colorectal cancer, were measured directly during clinical examination. Limitations include limited information on dietary risk or protective factors such as calcium and fiber intake, or non-dietary factors such as NSAIDs, hormone replacement therapy or other drug use.

In conclusion, our large-scale cohort study observed differential risk factor profiles according to subsites of colorectal cancer. Our findings support the hypothesis that there are differential risk factor profiles for subsites of colorectal cancer. Further researches on predicting colorectal cancer risk based on lifestyle risk factors require consideration of subsites of colon and rectum as different organs to ensure more precise estimation of risks.
